# Biomonitoring of arsenic, cadmium and lead in two artisanal and small-scale gold mining areas in Zimbabwe

**DOI:** 10.1007/s11356-021-15940-w

**Published:** 2021-08-19

**Authors:** Stefan Rakete, Given Moonga, Anna-Maria Wahl, Viola Mambrey, Dennis Shoko, Dingani Moyo, Shamiso Muteti-Fana, Myriam Tobollik, Nadine Steckling-Muschack, Stephan Bose-O’Reilly

**Affiliations:** 1 Institute and Clinic for Occupational-, Social- and Environmental Medicine, University Hospital, LMU Munich, Ziemssenstr. 1, D-80336 Munich, Germany; 2 Center for International Health, LMU University Hospital Munich, Munich, Germany; 3 Department of Epidemiology and Biostatistics, University of Zambia, Lusaka, Zambia; 4 Tailjet Consultancy Services, Harare, Zimbabwe; 5 School of Public Health, Faculty of Health Sciences, Occupational Health Division, University of the Witwatersrand, Johannesburg, Republic of South Africa; 6 Faculty of Medicine & Faculty of Social Sciences, Midlands State University, Gweru, Zimbabwe; 7 Department of Community Medicine, UZ College of Health Sciences, Harare, Zimbabwe; 8 Section Environmental Medicine and Health Effects Assessment German Environment Agency, Berlin, Germany; 9 Institute of Public Health, Medical Decision Making and Health Technology Assessment, Department of Public Health, Health Services Research and Health Technology Assessment, UMIT (Private University for Health Sciences, Medical Informatics and Technology), Hall in Tirol, Austria; 10 University Children’s Hospital Regensburg (KUNO-Clinics), Clinic St. Hedwig, University of Regensburg, Regensburg, Germany

**Keywords:** Biomonitoring, Toxic metals, Arsenic, Cadmium, Lead, Artisanal and small-scale gold mining, Zimbabwe

## Abstract

People living and working in artisanal and small-scale gold mining (ASGM) areas are frequently exposed to elemental mercury (Hg), which is used for gold extraction. However, additional exposure to other toxic metals such as arsenic (As), cadmium (Cd) and lead (Pb) may result from mining-related activities and could be ingested via dust, water or food. In these areas, only limited biomonitoring data is available for toxic metals other than Hg. In particular, data about the exposure to As, Cd and Pb is unavailable for the Zimbabwean population. Therefore, we conducted a cross-sectional study in two ASGM areas in Zimbabwe to evaluate the internal exposure to these metals. In total, urine and blood samples from 207 people that identified themselves as miners were collected and analysed for As and Cd in urine as well as Pb in blood by GF-AAS. Median levels (interquartile ranges in μg/l) of As and Pb were 9.7 μg/l (4.0, 18.5) and 19.7 μg/l (12.5, 34.5), respectively. The 25th percentile and the median for Cd were below the limit of detection (0.5 μg/l); the 75th percentile was at 0.9 μg/l. The results were compared to reference values found for the general population in the USA and Germany, and a significant number of participants exceeded these values (As, 33 %; Cd, 27 %; Pb, 32 %), indicating a relevant exposure to toxic metals. Although not representative for the Zimbabwean population, our results demonstrate that the exposure to toxic metals is relevant for the public health in Zimbabwe and requires further investigation.

**Supplementary Information:**

The online version contains supplementary material available at 10.1007/s11356-021-15940-w.

## Introduction

Artisanal and small-scale gold mining (ASGM) is predominantly an informal, poverty-driven, poorly resourced, comparatively inefficient and transient sector. Nonetheless, it provides an income to people in many developing countries rich in gold resources, and it is estimated that about 15 million miners are involved globally (Seccatore et al. 2014). ASGM in Zimbabwe has been growing very rapidly over the last 40 years. This growth was even faster over the last 2 decades, from an estimated 300,000 miners in 2000 to over 1.5 million today (Mkodzongi and Spiegel 2020, Mudzwiti et al. 2015). This has been due to a variety of factors including a persistently shrinking economy across multiple industries along with recurrent droughts during the last 2 to 3 decades. Consequently, there has been a growth in terms of tonnages mined and processed as well as gold produced. However, this resulted in the expansion of mining sites, increased used of process chemicals such as mercury and the exposure of new rock surfaces. This has led to the worsening of adverse environmental and public health impacts due to the release of toxic metals, mainly due the use of mercury (Hg) in the mining process (Billaud et al. 2004; Mudzwiti et al. 2015).

More than 90% of gold deposits in Zimbabwe are associated with the largely mafic/basic and cratonic greenstone belts, while the rest is located within the Limpopo Mobile Belt to the south and the Lomagundi meta-sediments in the north. The genesis of most gold deposits in Zimbabwe (>75%) involved the generation of superheated (>2000 °C) aqueous hydrothermal sulphide complexes at depth, in the upper mantle to lower crust. Gold is mainly transported in solution as aqueous complexes of hydrogen sulphides/bi-sulphides and chlorides as well as metal complexes (Foster 1985). Various elements, and especially heavy metal elements, have an affinity for dissolved hydrogen sulphides and chlorides in hydrothermal ore-forming solutions. This includes, but is not limited to, As, silver (Ag), Cd, Hg, Pb, selenium (Se), antimony (Sb), tellurium (Te), thallium (Tl) and zinc (Zn). These metals and many others are commonly associated with gold ores and are routinely used as pathfinders in gold exploration geochemistry (Saunders et al. 2014). Especially As, Cd and Pb are relatively toxic compared to other metals and were categorized within the top ten chemicals of public health concern by the World Health Organization (International Programme on Chemical Safety (IPCS) 2010). Mining activities such as excavation, crushing and milling, which are used in ASGM, may result in the increased liberation of these toxic metals. While the precious gold is collected at the end of the mining process, the other metals may end up in the tailings dumps at mining locations and thus represent an exposure hazard for people living and working in these mining areas.

Although, biomonitoring of As (Adu-Poku et al. 2019; Basu et al. 2011; Nyanza et al. 2019; Obiri et al. 2016), Cd (Basu et al. 2011; Obiri et al. 2016) and Pb (Gottesfeld et al. 2019; Nyanza et al. 2019; Obiri et al. 2016) has been conducted in several ASGM areas, exposure data is still relatively limited, particularly in Zimbabwe. Therefore, the purpose of this study was the analysis of As and Cd in urine as well as Pb in blood samples collected during a cross-sectional study involving people that identified themselves as miners in two ASGM areas in Zimbabwe. Data on Hg levels and health-related quality of life from this study were published elsewhere (Butscher et al. 2020; Mambrey et al. 2020; Wahl et al. 2021).

## Materials and methods

### Study design

This cross-sectional study was conducted within 2 weeks in March 2019 at two hospitals in the gold mining towns of Kadoma and Shurugwi Districts (Zimbabwe), respectively. Inclusion criteria for participation were a minimum age of 18 years. All females and males that identified themselves as miners and worked for at least a month were included. There were no specific exclusion criteria other than age. Participants were recruited using snowball sampling where participants recruit further participants among their colleagues. This sampling technique was used due to a widespread and hard-to-reach target population. Each participant signed an informed consent form and material transfer agreement, prior to the data and sample collection. All documents were available in the three main languages English, Shona and Ndebele spoken in Zimbabwe. Altogether, 207 participants consented to participate (131 from Kadoma and 76 from Shurugwi). Participation in the study was voluntary. Participants were asked to fill out questionnaires concerning general information on demographics. To ensure a confidential analysis, the samples and data were pseudonymized. More information about the study was previously published (Mambrey et al. 2020).

### Urine and blood collection

All sample containers were labelled with the participant’s code for future allocation. Spot urine samples were collected using disposable urine collection cups. For transport and analysis, aliquots of the urine samples were transferred into a Urine Monovette (Sarstedt®). To prevent bacterial growth and degradation, the samples were acidified with nitric acid to a pH of approximately 2, which was tested with a pH strip. Trained health professionals took venous blood samples into 7 ml lithium-heparin-coated tubes for trace metal analyses (Sarstedt®) from all participants. All samples were continuously stored at 4 °C. Once located in the laboratory, samples were stored at –18 °C until analysis.

### Analysis of trace metals in urine and blood

All samples were at least analysed in duplicate after thawing on a roll mixer. For quality control (QC), certified reference materials (ClinChek®, Recipe, Munich, Germany) for whole blood and urine were analysed daily, and the sample analysis was only continued if the QC results were within the given specifications. As, Cd and Pb were analysed by graphite furnace atomic absorption spectroscopy (GF AAS, AAnalyst 600, Perkin Elmer, Rodgau, Germany) with the furnace programmes according to the recommendations of the manufacturer. The individual specifications for each element can be found below. The quantitation of all elements based on the standard addition method and the limit of detection (LOD) was calculated from the blank signal.

#### Analysis of total As in urine

Total As in urine was analysed at a detection wavelength of 193.7 nm. Urine samples were diluted sixfold with 0.01% Triton-X in 0.13% nitric acid. Twenty microlitres of this dilution were automatically pipetted into the graphite tube of the GF-AAS. Five micrograms of Pd (as Pd(NO_3_)_2_) and 3 μg Mg(NO_3_)_2_ were added as matrix modifiers. For standard addition, 10 and 20 pg As were directly added to the sample in the graphite tube, respectively. The LOD was at 0.5 μg/l.

#### Analysis of Cd in urine

Cd in urine was analysed at a detection wavelength of 228.8 nm. Urine samples were diluted fourfold with 0.01% Triton-X in 0.13% nitric acid. Twenty microlitres of this dilution were automatically pipetted into the graphite tube of the GF-AAS. Fifty micrograms of NH_4_H_2_PO_4_ and 3 μg Mg(NO_3_)_2_ were added as matrix modifiers. For standard addition, 0.5 and 1 pg Cd were directly added to the sample in the graphite tube, respectively. The LOD was at 0.5 μg/l.

#### Analysis of Pb in blood

Pb in blood was analysed at a detection wavelength of 193.7 nm. Blood samples were diluted tenfold with 0.05% Triton-X. Twenty microlitres of this dilution were automatically pipetted into the graphite tube of the GF-AAS. Fifty micrograms of NH_4_H2PO_4_ and 3 μg Mg(NO_3_)_2_ were added as matrix modifiers. For standard addition, 5 and 10 pg Pb were directly added to the sample in the graphite tube, respectively. The LOD was at 1.0 μg/l.

### Analysis of creatinine in urine

Creatinine in urine samples was determined for creatinine-corrected levels of toxic metals in urine. Creatinine-corrected urine values were considered in order to account for the influence of the effect of urine dilution on the exposure indicator. Urine samples were sent to the central laboratory of University Hospital of LMU and analysed with Cobas C702 using the Jaffé method. Creatinine-corrected values from urine samples with creatinine levels < 0.3 g/l and > 3.0 g/l were excluded from statistical analysis.

### Statistical analysis

All data analyses were performed with SPSS (version 26, IBM). One participant was excluded from statistical analysis, as the urine sample apparently contained blood. For samples below the LOD, the result was set to ½ LOD for further statistical analysis. Descriptive analysis included the geometric mean, minimum, maximum, median, 25th percentile, 75th percentile and 95th percentile. Differences in toxic metal concentrations between groups (gender, living area and self-reported fish consumption) were tested using the Mann-Whitney *U* test. Continuous variables (toxic metals, age, area years and mining years) were correlated using the Spearman correlation. Results for Hg in blood in urine and blood from the same participants used for correlation analyses were previously published elsewhere (Mambrey et al. 2020).

## Results

Demographic information on the study population can be found in Table 1. One sample had to be excluded due to obvious sample contamination. Consequently, the levels of As and Cd in urine as well as the levels of Pb in blood were analysed for 206 participants, and the results are given in Table 2. Although urine samples with creatinine levels < 0.3 or > 3.0 g/l should preferably not be used for biomonitoring, we decided to only refrain from creatinine correction, as sampling could not be repeated (Cocker et al. 2011). Therefore, thirteen urine samples with urinary creatinine levels outside of this range were excluded from creatinine correction. The participants’ levels of As in urine and Pb in blood follow a log-normal distribution (Figure S1), which was expected for this population. For Cd in urine, a log-normal distribution is anticipated, too. However, the majority of samples were below the LOD. The results were further stratified by gender (82 % male), living area (63% Kadoma) and self-reported fish consumption (80 % at least once a week), and differences were tested for significance (Table S1). For gender, urinary As levels were significantly higher in women. In contrast, blood Pb levels were significantly higher in men. For the area of living, significantly higher levels of As in urine were found for Kadoma if corrected for urinary creatinine. For self-reported fish consumption, no significant differences were found for any toxic metal.

**Table 1:** Demographic information on the study population

Age	*N*	207	
	Median (min.–max.)	38 (18–77)	
		***N***	**(%)**
Gender	Males	169	(81.6)
	Females	38	(18.4)
Living area	Kadoma	131	(63.3)
	Shurugwi	76	(36.7)
Fish consumption	< once a week	42	(20.3)
	> once a week	165	(79.7)

**Table 2:** Descriptive analysis of biomonitoring results for urinary levels of As and Cd and blood levels of Pb. Creatinine correction was not applied for urine samples with creatinine levels below 0.3 or above 3.0 g/l. Results were compared to international reference and threshold values for As and Cd in urine and Pb in blood, and the percentage of exceedances in this study is given in the brackets. Reference values represent the actual internal exposure of a representative population. Threshold values were derived from toxicological data. *NHANES* National Health and Nutrition Examination Survey, USA, *UBA* German Environment Agency, *CDC* Centers for Disease Control and Prevention, USA, *NIOSH* National Institute for Occupational Safety and Health, USA, *HBM-II* human biological monitoring alert level (Centers of Disease Preventions and Control (CDC) 2019, Schulz et al. 2011, UBA - German Environment Agency 2019)

	Creatinine in urine	As in urine		Cd in urine		Pb in blood
	g/l	μg/l	μg/g crea.	μg/l	μg/g crea.	μg/l
N	206	206	193	206	193	206
LOD	0.1	0.5		0.5		1
< LOD	0	12		120		0
GM	1,3	7.2	5.6	0.6	0.4	21.9
Minimum	0.1	< LOD		< LOD		6.6
25th percentile	1.0	3.7	2.7	< LOD		12.5
Median	1.4	9.7	6.5	< LOD		19.7
75th percentile	2.0	17.1	13.2	0.9	0.7	34.1
95th percentile	2.8	47.1	33.6	3.6	1.8	76.4
Maximum	4.6	460.5	250.3	11.4	4.9	275.7
		As in urine [μg/l]		Cd in urine [μg/l]		Pb in blood [μg/l]
Reference values (exceedances in %)						
NHANES		49.9 (3.6)		1.1 (22)		28.9 (32)
UBA		15.0 (33)		0.8 (27)		30 (♀, 24 %) 40 (♂, 20 %)
Threshold values (exceedances in %)						
NIOSH (CDC)		n.a.		n.a		50 (11)
UBA (HBM-II)		n.a.		4.0 (4)		n.a.

*LOD* limit of detection, *< LOD* number of results below limit of detection, *GM* geometric mean, *n.a.* not available

For further evaluation, the results were compared to available international reference and threshold values from Germany and the USA (Centers of Disease Preventions and Control (CDC) 2019, Schulz et al. 2011, UBA - German Environment Agency 2019). The reference and threshold values for As, Cd and Pb as well as the percentage of participants that exceeded these values are given in Table 2. The 95th percentiles of Cd and Pb found in this study were higher than the reference values of the NHANES and UBA data. For As in urine, the 95th percentile found in this study was higher than the value proposed by UBA but comparable to NHANES.

The results for the correlation between toxic metals (including Hg), age, area years and mining years are given in Table S2. In general, correlation of the biomonitoring results with age, area years and mining years was found to be very low. However, Hg levels in urine and blood showed a weak positive correlation with mining years. In contrast, Cd in urine showed a weak negative correlation with age. If the levels of toxic metals were correlated to each other, a strong positive relationship was found for non-corrected and corrected levels of As, Cd and Hg in urine, respectively. Furthermore, a strong correlation was found for Hg in urine and Hg in blood. Whereas As, Cd and Pb mainly showed no to very weak correlation among each other, Hg levels in urine and blood showed a relatively moderate positive correlation with As, Cd and Pb.

## Discussion

As explained in the introduction, toxic metals may be associated with gold-containing ores in ASGM areas. Therefore, it seems plausible that elevated As, Cd and Pb levels in the participants are related to the mining activities in Kadoma and Shurugwi. Below, we discuss the results for the individual metals and compare them to previously published studies in mining areas (Table 3). Although some of the studies found comparable results, the exposure to toxic metals likely depends on multiple factors such as the study population, the local concentrations of metals in the ore, the diet and many others. Generally, living close to mining areas seems to have a significant effect on the body burden (Basu et al. 2010, Molina-Villalba et al. 2015, Nemery and Banza Lubaba Nkulu 2018, Obiri et al. 2016). One possible explanation is that contaminated mining tailings contribute to the exposure to toxic metals by causing elevated concentrations in water, food and airborne dust (Moreno et al. 2010; van Straaten 2000).

**Table 3:** Comparison of the study results (all values are given in μg/l) with other studies in mining areas. All values are given as 25th percentile (P25), median and 75th percentile (P75) unless marked otherwise (^#^)

Parameter [μg/l]	Values measured in this Study	Values measured in studies in current and former mining areas (country)	Reference
	P25–median–P75	P25–median–P75	
As in urine	4.0–10.0–18.5	4.9–9.4–15.1 (Tanzania)^1^	Nyanza et al. (2019)
		73.2–100.2–135.3 (Ghana)	Basu et al. (2011)
		11.1–16.5–19.4 (Mexico)^2^	Moreno et al. (2010)
		0.5–1.17–1.93 (Spain)^2^	Molina-Villalba et al. (2015)
		0.06 (Guatemala)	Basu et al. (2010)
Cd in urine	< LOD–< LOD–0.9	0.25–0.36–0.6 (Ghana)	Basu et al. (2011)
		0.13–0.29–0.46 (Spain)^2^	Molina-Villalba et al. (2015)
		0.11 (Guatemala)	Basu et al. (2010)
Pb in blood	12.5–19.9–34.5	16.9–25.4–33.7 (Tanzania)^1^	Nyanza et al. (2019)
		64–94–113 (Mexico)^2^	Moreno et al. (2010)
		26.7 (Guatemala)	Basu et al. (2010)
		13.0 (Zambia)	Yabe et al. (2020)
		21.5 (Nigeria)	Gottesfeld et al. (2019)
		28.0 (Ghana)^#^	Obiri et al. (2016)

^1^Pregnant women

^2^Children

^#^Mean

*LOD* limit of detection

### Arsenic

In contrast to the other metals, the reference values for As in urine from Germany (UBA) and the USA (NHANES) differ considerably from one another (15.0 vs 49.9 μg/l). This is likely due to different exposures to As, e.g. by fish consumption and other sources such as drinking water. However, reference values represent the actual exposure in the general population and cannot be used for toxicological evaluation. Consequently, the number of participants in this study that were above reference values for As heavily depends on which value will be used. It seems that the exposure to As is more comparable to the US population. However, we did not analyse the As species for differentiate inorganic (As(III), As(V)) and organic (e.g. arsenobetaine) As species. A major source of organic As is fish. However, self-reported fish consumption had no effect on As levels in urine. Zimbabwe has no access to open sea. Consequently, the consumed fish is commonly freshwater fish which usually contains relatively low amounts of As. The elevated levels of As in women and participants from Kadoma may be explained by different dietary patterns or a generally elevated exposure to As due to mining activities. Our results were comparable to what has been found in an ASGM area in Tanzania, but also in non-active mining areas in Mexico (Moreno et al. 2010; Nyanza et al. 2019). However, Basu et al. found a tenfold higher median As level in a Ghanaian ASGM study (Basu et al. 2011). In contrast, studies from Spain and Guatemala found relatively low levels of As in the urine (Basu et al. 2010; Molina-Villalba et al. 2015).

### Cadmium

Most of the samples were below the LOD, indicating a generally low exposure. Still, a considerable number of participants were above the references and threshold values. Cd levels are generally elevated in smokers. Unfortunately, we do not have the data for smoking in our study. In Zimbabwe’s mining areas, far more men smoke compared to women (Billaud et al. 2004). However, this was not reflected in our results, where women were more frequently above international reference and threshold values for Cd (data not shown). For Cd in urine, the studies from Ghana, Guatemala and Spain showed very similar results (Basu et al. 2010; Basu et al. 2011; Molina-Villalba et al. 2015). Therefore, Cd exposure in ASGM and other mining areas seems to be relatively low compared to other toxic metals.

### Lead

About a third of the participants were above international reference values. The exposure to lead could be via contaminated drinking water, although the uptake of Pb in the gastrointestinal tract of adults is considered relatively low and airborne dust due to mining activities. Besides this, Pb exposure could be due to emissions of leaded gasoline, which was used longer in Africa than in the US or Europe (Todd and Hazel 2010). Therefore, the recent exposure to these emissions might still be reflected by elevated blood Pb levels. The results of this study are comparable to what has been found in other studies (Basu et al. 2010; Gottesfeld et al. 2019; Obiri et al. 2016; Yabe et al. 2020). However, in a study from Mexico, blood Pb levels were significantly higher (Moreno et al. 2010).

## Limitations

Unfortunately, we did not have access to information about smoking, diet other than fish and levels of toxic metals in water, food, soil and air, thus limiting a more thorough exposure assessment. Additionally, we were not able to speciate As due to technical and financial limitations, further limiting exposure assessment and comparison with other studies. For As and Cd, the sensitivity of the analytical method was too low for some samples. In fact, Cd could not be detected in more than half of the samples, clearly limiting Cd exposure assessment. Nevertheless, the LOD was below the used reference and threshold levels. However, a relatively high number of participants were above international reference values (22 % > NHANES, 27 % > UBA). This may be explained by the fact that the Cd levels of 51 samples were relatively close to the LOD. Consequently, these results may be subject to some uncertainty regarding absolute quantitation. Furthermore, the inclusion of a control group from a non-mining area in Zimbabwe may have provided information if the exposure to As, Cd and Pb is actually caused by mining activities. However, this study was designed as a cross-sectional study as many studies had demonstrated that mining activities are associated with increased exposure to toxic metals.

## Conclusions

This is, to the best of our knowledge, the first study that analysed the urinary levels of As and Cd as well as the blood levels of Pb in people identifying themselves as artisanal and small-scale gold miners in Zimbabwe. A high proportion of the participants had As, Cd and Pb levels above international reference levels. Therefore, the exposure to toxic metals in the two ASGM areas in Zimbabwe is relevant to public health and should be the subject of further investigation to clarify the influence of possible confounders, e.g. the diet. Furthermore, the exposure to toxic metals should be assessed in the general population of Zimbabwe to investigate if the results found in this study are related to ASGM activities.

## Supplementary information

ESM 1 (DOCX 61 kb)

## Data Availability

The datasets used and/or analysed during the current study may be made available from the corresponding author on reasonable request.
