# Supplementary material for: Biomonitoring of arsenic, cadmium and lead in two artisanal and small-scale gold mining areas in Zimbabwe
Source: Environ Sci Pollut Res Int. 2021 Aug 19;29(3):4762–8. doi: 10.1007/s11356-021-15940-w (PMC8741681; doi:10.1007/s11356-021-15940-w)

**Supplemental Information:**

Table S1: 25^th^ percentile, (P25), median (P50) and 75^th^ percentile (P75) levels of urinary levels of creatinine, As and Cd and blood levels of Pb stratified by gender, area and self-reported fish consumption. Creatinine correction was not applied for urine samples with creatinine levels below 0.3 or above 3.0 g/l. Differences between the groups were tested with Mann-Whitney-U test.

|  |  | **creatinine in urine** | | | **As in urine** | | | | | | **Cd in urine** | | | | | | **Pb in blood** | | |
| --- | --- | --- | --- | --- | --- | --- | --- | --- | --- | --- | --- | --- | --- | --- | --- | --- | --- | --- | --- |
|  | **n (*)** | **g/l ^#^** | | | **µg/l** | | | **µg/g crea.** | | | **µg/l** | | | **µg/g crea.** | | | **µg/l** | | |
|  |  | **P25** | **P50** | **P75** | **P25** | **P50** | **P75** | **P25** | **P50** | **P75** | **P25** | **P50** | **P75** | **P25** | **P50** | **P75** | **P25** | **P50** | **P75** |
| **Gender** |  |  |  |  |  |  |  |  |  |  |  |  |  |  |  |  |  |  |  |
| **Female** | 37 (0) | 1.1 | 1.4 | 1.9 | 5.7 | 17.8 | 29.9 | 4.4 | 11.7 | 22.4 | < LOD | | 1.5 | < LOD | | 0.9 | 9.1 | 16.3 | 29.8 |
| **Male** | 169 (13) | 1.0 | 1.4 | 2.0 | 3.2 | 8.3 | 16.7 | 2.7 | 5.9 | 11.2 | < LOD | | 0.9 | < LOD | | 0.6 | 13.9 | 20.5 | 35.2 |
| **p** | 0.757 | 0.757 | | | 0.001 | | | 0.004 | | | 0.411 | | | 0.618 | | | 0.015 | | |
|  |  |  |  |  |  |  |  |  |  |  |  |  |  |  |  |  |  |  |  |
| **Area** |  |  |  |  |  |  |  |  |  |  |  |  |  |  |  |  |  |  |  |
| **Kadoma** | 130 (8) | 1.0 | 1.4 | 1.9 | 4.2 | 10.9 | 18.1 | 3.2 | 7.7 | 13.7 | < LOD | | 0.9 | < LOD | | 0.7 | 13.8 | 20.6 | 34.9 |
| **Shurugwi** | 76 (5) | 1.0 | 1.6 | 2.2 | 2.4 | 7.9 | 16.7 | 1.7 | 5.1 | 11.2 | < LOD | | 0.8 | < LOD | | 0.6 | 11.7 | 18.4 | 28.9 |
| **p** |  | 0.105 | | | 0.110 | | | 0.024 | | | 0.830 | | | 0.498 | | | 0.197 | | |
|  |  |  |  |  |  |  |  |  |  |  |  |  |  |  |  |  |  |  |  |
| **fish consumption** |  |  |  |  |  |  |  |  |  |  |  |  |  |  |  |  |  |  |  |
| **less than once a week** | 41 (1) | 0.8 | 1.5 | 2.0 | 1.7 | 9.7 | 16.5 | 1.8 | 6.0 | 12.5 | < LOD | | 1.0 | < LOD | | 0.5 | 11.6 | 18.2 | 30.5 |
| **at least once a week** | 164 (12) | 0.9 | 1.4 | 2.0 | 3.7 | 9.7 | 18.0 | 3.0 | 6.5 | 13.2 | < LOD | | 0.9 | < LOD | | 0.7 | 13.0 | 20.6 | 34.2 |
| **p** |  | 0.919 | | | 0.597 | | | 0.390 | | | 0.819 | | | 0.882 | | | 0.220 | | |

* excluded from creatinine correction, ^#^ includes all creatinine results, LOD: limit of detection for Cd was 0.5 µg/l

Table S2: Correlation between the different toxic metals and sociodemographic parameters (Spearman's correlation).

|  | **Unit** | **As in urine** | | **Cd in urine** | | **Hg in urine** | | **Hg in blood** | **Pb in blood** |
| --- | --- | --- | --- | --- | --- | --- | --- | --- | --- |
|  |  | **µg/l** | **µg/g crea** | **µg/l** | **µg/g crea** | **µg/l** | **µg/g crea** | **µg/l** | **µg/l** |
| **Age** | **years** | -0.033 | -0.008 | -0.129 | **-0.148*** | -0.088 | -0.096 | -0.062 | -0.098 |
| **Area years** |  | -0.092 | -0.118 | -0.071 | -0.057 | 0.022 | 0.028 | -0.031 | -0.054 |
| **Mining years** |  | 0.001 | -0.011 | -0.098 | -0.082 | **0.176^*^** | **0.191^**^** | **0.145^*^** | 0.045 |
| **As in urine** | **µg/l** |  | **0.914**** | **0.203**** | -0.029 | **0.243**** | 0.130 | **0.137*** | 0.015 |
|  | **µg/g crea** |  |  | 0.034 | 0.038 | 0.135 | 0.138 | **0.165*** | 0.024 |
| **Cd in urine** | **µg/l** |  |  |  | **0.788**** | **0.322**** | **0.260**** | **0.179**** | 0.012 |
|  | **µg/g crea** |  |  |  |  | **0.248**** | **0.308**** | **0.280**** | 0.051 |
| **Hg in urine** | **µg/l** |  |  |  |  |  | **0.967**** | **0.743**** | **0.230**** |
|  | **µg/g crea** |  |  |  |  |  |  | **0.778**** | **0.247**** |
| **Hg in blood** | **µg/l** |  |  |  |  |  |  |  | **0.237**** |

** p<0.01, * p<0.05

Figure S1: Histograms of As and Cd levels in urine and Pb levels in blood.


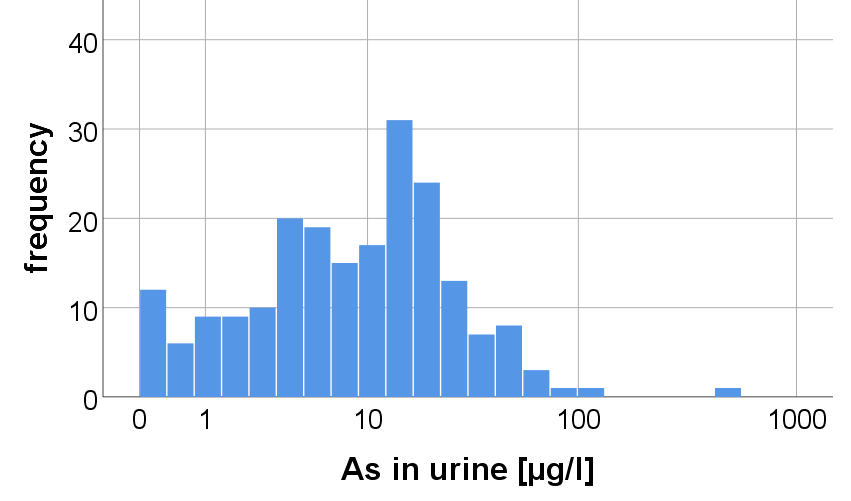


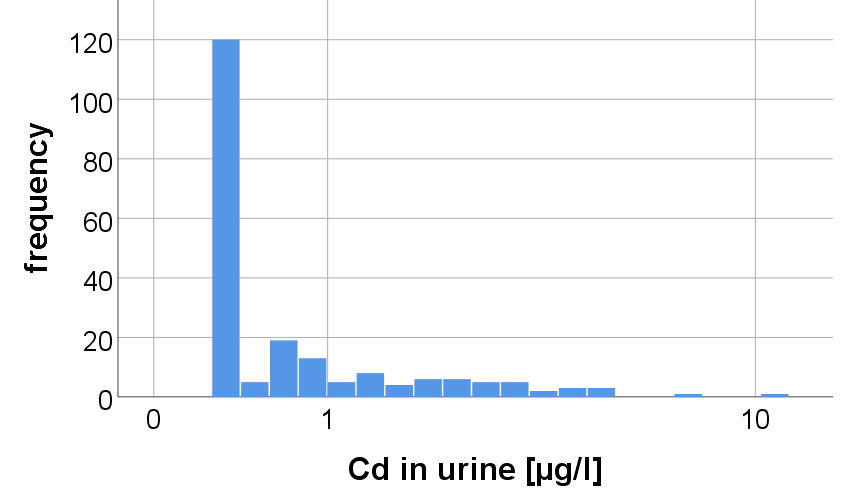


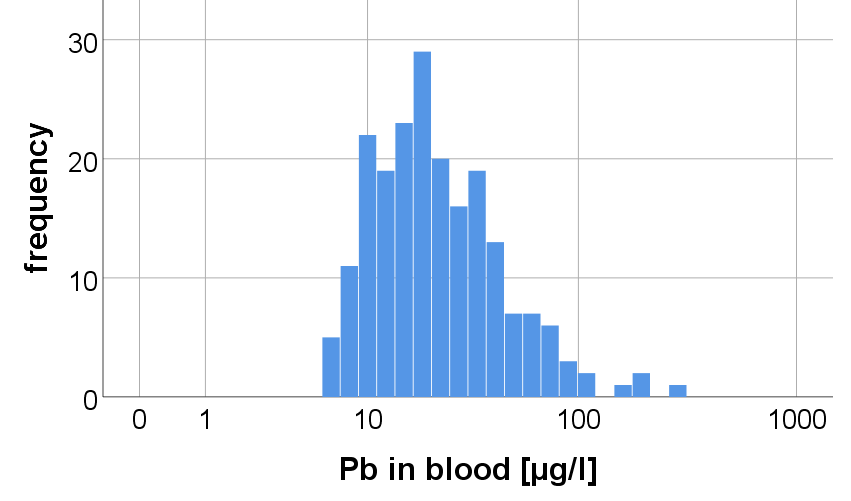

Supplement: Supplementary file 1 — (DOCX 61 kb) [file 11356_2021_15940_MOESM1_ESM.docx]
